# Screening of the mitochondrial A1555G mutation in patients with sensorineural hearing loss

**DOI:** 10.1016/S1808-8694(15)31384-7

**Published:** 2015-10-17

**Authors:** Luciano Pereira Maniglia, Bruna Carolina Lemos Moreira, Magali Aparecida Orate Menezes da Silva, Vânia Belintani Piatto, José Victor Maniglia

**Affiliations:** 1 Master’s degree student, faculty member of the Otorhinolaryngology and Head & Neck Surgery Department, Medical School, S. J. Rio Preto, SP, FAMERP; 2 Medical student, Medical School, São José do Rio Preto, SP, FAMERP; 3 Master’s degree; head of the Speech Therapy Unit, Otorhinolaryngology and Head & Neck Surgery Department, Medical School, S. J. Rio Preto, SP, FAMERP; 4 Doctor, Adjunct professor; 5 Livre-docente (habilitation) professor, head of the Otorhinolaryngology and Head & Neck Surgery Department, Medical School, S. J. Rio Preto, SP, FAMERP. Medical School, São José do Rio Preto, SP, FAMERP

**Keywords:** aminoglycosides, hearing loss, mitochondrial DNA, mutation

## Abstract

The A1555G mitochondrial mutation is the main alteration associated with aminoglycoside-induced deafness.

**Aim:**

to investigate the prevalence of the A1555G mutation in patients sensorineural hearing loss patients with and without aminoglycosides antibiotic use.

**Material and Method:**

a study of 27 cases with deafness as the sample, and 100 neonates with normal hearing as the control group. DNA was extracted from blood leukocyte samples, and specific oligonucleotide primers were designed to amplify the cytochrome b gene and the region which encloses the A1555G mutation of the mitocondrial DNA using the polymerase chain reaction and restriction fragment length polymorphism.

**Design:**

a cross-sectional case study.

**Results:**

a region of the cytochrome b gene was amplified and the presence of the mtDNA was confirmed in all of the 127 cases. The A1555G mutation was not found in any of the 27 patients with hearing loss or the control group with 100 neonates.

**Conclusion:**

the results agree with studies stating that the A1555G mutation is not prevalent in the Americas. There is interest in establishing the real prevalence of this mutation and to investigate other mutations that may cause hearing loss, associated or not with the use of aminoglycosides, in the Brazilian population.

## INTRODUCTION

It is estimated that in developed countries, one child in 750 births presents sensorineural hearing loss, and that one child in each 1,000 births becomes deaf before adulthood. The prevalence increases further and reaches 50% in octogenarians.^1^ About 60% of all causes of prelingual hearing loss may be attributed to hereditary factors. Thus, inheritance is becoming more and more relevant in hearing loss and/or deafness. The remaining 40% are caused by various etiologies.^2^ There are three identifiable possible inheritance patterns in hereditary hearing loss: autosomal recessive, autosomal dominant and X-linked. About 75% to 85% of non-syndromic prelingual hearing loss cases manifest as autosomal recessive conditions. Autosomal dominant forms respond for about 15% to 25% of cases; the remaining 1% to 3% cases are Mendelian inheritance X-linked conditions. There are forms inherited exclusively from the mother - mitochondrial inheritance - in about 0.5% to 1% of sensorineural hearing loss of genetic causes; spontaneous mutation may also occur.^2^, ^3^ A relation between mitochondrial disease and hearing loss was established in 1986 based on the study of a patient with mitochondrial myopathy and hearing loss.^4^

Mitochondrial mutations, especially those in the 12S rRNA and tRNASer(UCN) genes, are important causes of non-syndromic sensorineural hearing loss in some population groups.^5^ Among these mutations, a substitution of A->G nitrogenated bases in position 1555 of the mitochondrial DNA 12S rRNA gene is or particular interest as the main cause of aminoglycoside-induced hearing loss. It was identified in 1993 by a study that described it as a mitochondrial rRNA mutation that led to non-syndromic hearing loss; this was the first molecular genetic analysis of aminoglycoside-induced ototoxicity.^6^

Many studies have suggested that the A1555G mutation by nitrogenated base substitution generates a new pair of C-G bases, making the secondary structure of the human 12S rRNA gene strictly similar to the corresponding region of the E. coli 16S rRNA gene; this is referred to as the decoding bacterial rRNA region and an important action site for aminoglycosides.^6^, ^7^ Aminoglycoside binding to the decoding region results in protein translation errors and ensuing bacterial death. When there is a generally homoplasmic mutation altering the human 12S rRNA gene, making its structure similar to that of the bacterial rRNA, aminoglycosides concomitantly bind to cochlear cell mitochondrial DNA, which potentializes its toxic effect in the inner ear, resulting in hearing loss. These studies, therefore, demonstrate the highly specific recognition mode among RNA molecules and aminoglycosides.^7^

In contrast with systemic mitochondrial mutations, which usually affect only a fraction of all mitochondrial DNA molecules (heteroplasmia), mutations associated with non-syndromic sensorineural hearing loss are generally homoplasmic, and phenotypes may differ considerably among members of the same family, ranging from deep hearing loss to normal hearing. Many factors, such as nuclear genes, mitochondrial DNA haplotypes, environmental factors or tissue-specific factors, may operate independently or in association, and may alter the clinical expression. However, research has not found nuclear modifying genes or correlations among mutations and mitochondrial haplotypes. The only known environmental factors affecting the A1555G mutation are the aminoglycosides.^8^

Although mitochondrial mutations are etiologically relevant in sensorineural hearing loss, few studies have been made in developing countries, especially in Brazil. The purpose of this study, therefore, was to investigate the prevalence of the mitochondrial A1555G mutation in patients with non-syndromic sensorineural hearing loss with and with no exposure to aminoglycosides.

## SERIES AND METHOD

A cross-sectional study was made between September and October 2006 of 20 index cases of non-syndromic sensorineural hearing loss (12 males and 8 females) aged from 1 to 37 years, previously submitted to GJB2 gene and D(GJB6-D13S1830) mutation molecular analysis; the cases had no molecular changes.^9^ The index cases in our sample consisted of 16 sporadic cases (single cases within a family) and four familiar cases (familiar cases). Relatives with hearing loss were also assessed (2 males and 5 females) aged from 5 to 45 years. Thus, 27 hearing loss patients were studied.

A complete history was taken from each patient to investigate the age of onset of hearing loss, other cases in the family, the use of ototoxic drugs (aminoglycosides), and to exclude other environmental causes such as maternal-fetal infections, perinatal complications, meningitis, acoustic trauma and consanguineous marriages. Patients underwent a physical examination (systemic and otorhinolaryngological) and laboratory exams, to exclude syndromic forms of hearing loss (especially craniofacial dimorphism, skin alterations, and branchial, heart and thyroid anomalies, vision disorders, etc.). Other tests were an ophthalmologic evaluation (including fundoscopy), vestibular tests, and temporal bone computed tomography. Thus, a complete clinical assessment was made to exclude patients with hearing loss due to environmental factors except for aminoglycoside use, consanguinity, congenital inner ear malformations and genetic syndromes. Audiological testing consisted of pure tone audiometry, based on which patients with non-syndromic sensorineural hearing loss were included in the study and classified as mild (25–40 dB), moderate (41–60 dB), severe (61–80 dB) or profound (>81 dB).^10^

The patients in the sample were identified as the Hearing Loss Group (HLG) and divided into two subgroups:

HLG group I patients that used aminoglycosides (n=13).
HLG group II patients that did not use aminoglycosides (n=14).

One hundred term neonates with one-minute Apgar scores ≥7 and normal otoacoustic emissions tests (done within three days of birth) were selected as the control group (CG).

The Research Ethics Committee of our institution approved this study (protocol nº 2784/2006). Parents or legal caretakers read and signed a free informed consent form, after which 4.0 ml of whole blood (from a peripheral vein in the HLG group and from the umbilical cord after its ligature in the control group) was taken and placed on a VacutainerÒ tube containing anticoagulant (EDTA). Genomic DNA was extracted from the blood samples of both groups using the GFXTM Genomic Blood DNA Purification Kit (Amersham Pharmacia Biotech Inc.) according to the manufacturer’s instructions. Mitochondrial DNA fragments including the mutation region were amplified using the polymerase chain reaction (PCR) technique in a thermocycler (Applied Biosystems - GeneAmp PCR System 9700Ò) to detect the A1555G mutation.^11^ A pair of primers were synthesized for this reaction and were used to incorporate the deoxynucleotide triphosphates (dNTPs) to initiate DNA amplification, respecting base complementarity (A-T/C-G); mitochondrial DNA was then amplified. Oligonucleotide primer sequences and the conditions for the PCR were in accordance with the literature.^11^

A 643 bp fragment was amplified as a PCR product and then submitted to restriction fragment length polymorphism (RFLP) analysis, using the BsmAI^11^ enzyme (New England Biolabs)Ò during two hours at 55ºC. Enzymatic digestion of normal A1555G mutation sample fragments yields two fragments (413 bp and 230 bp) as a results of BsmAI enzyme restriction site recognition. Mutation samples yield only the 643 bp fragment, as there is no enzyme site recognition, due to substitution of A->G nitrogenated bases in position 1555 of the mitochondrial DNA.

A second primer pair was also synthesized to amplify a specific cytochrome b region.^12^ This specific region - a highly conserved area of the mitochondrial genome - is used as an internal amplification control to verify the presence of mitochondrial DNA in the samples. The expected size of PCR-amplified fragments using the CitbF and CitbR primers was 161 bp. Oligonucleotide primer sequences and the conditions for PCR were in accordance with the literature.^12^

Both primer pairs used in the PCR include regions in the “Human Mitochondrial DNA Revised Cambridge Reference Sequence.”^13^

The products of both reactions (PCR and RFLP) were analyzed using 2% agarose gel electrophoresis in a TBE 1X buffer containing ethidium bromide, at 0.5mg/mL concentration, submitted to ultraviolet light, to confirm the success of the reaction and gel; photographic documentation was done.

### Statistical analysis

A pilot study was done to assess 100 newborns to estimate the proportion (p) of probable A1555G mutation carriers in the initial sample. The statistical formula “sample size with a known population” was applied to the proportion (p) to obtain the final sample size (n) needed for statistically representing the total population (Np) of newborns born within the study period (Np=352 neonates). The final sample (n) was calculated using the abovementioned statistical formula with the following parameters: p=0.00 (estimated by the pilot sample); q=1.00; zc=3.00 (99.74% reliability); e=0.03 (3% estimated error); Np=352 (population size within the study period).

Formula: n = zc^2^ × p × q × N_p_

e^2^ × (Np - 1) zc^2^ × p × q

Results were expressed as percentages.

## RESULTS

PCR was done for amplification of the b cytochrome gene region to visualize the corresponding 161 bp fragment to confirm the presence of mitochondrial DNA in all 127 samples of the study - 100% (HLG I and II groups - n=27 and the control group - n=100).

PCR enabled amplification of the mitochondrial DNA 643 bp fragment encompassing the mutation area in all of the samples (100%) of HLG I and II groups (n=27) and the control group (n=100).

The A1555G mitochondrial mutation was not found in the study sample (100%); 413 bp and 230 bp fragments were found after enzymatic digestion, which were amplified in the absence of mutation in all samples within both groups (HLG I and II groups - n=27 and the control group - n=100).

**Chart 1:**
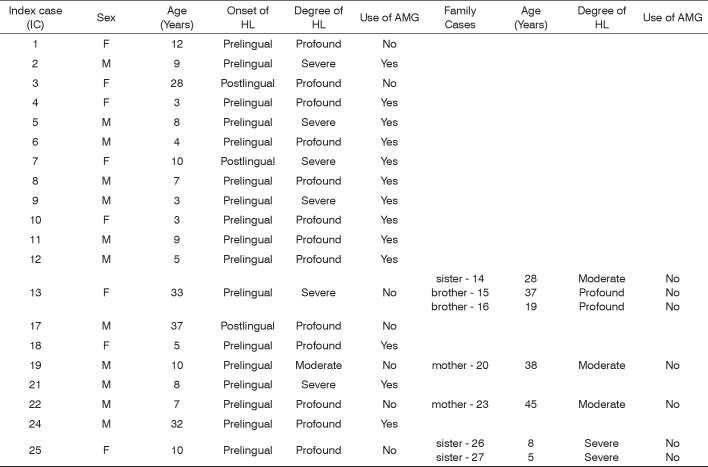
Clinical and audiometric data of the 20 index cases in the sample and the 7 affected family members (n=27) that underwent molecular analysis for the detection of the A1555G mitochondrial mutation.

Clinical and audiometric data from the index cases, such as sex, age, onset and degree of hearing loss, use of aminoglycosides and family cases are shown in Chart 1.

## DISCUSSION

Research in population groups in various European countries has found that conexin 26 (Cx26) gene mutations may cause hearing loss in a significant proportion of cases. Continued research has led to the discovery of other mutations, which has made possible molecular etiological identification in many patients; thus, early rehabilitation and genetic counseling have become feasible in these cases. It is interesting to note that the frequency of genes and mutations that cause hearing loss varies significantly among populations. The 35delG mutation in the Cx26 gene, for example, is highly prevalent among deaf persons of European descent,^9^, ^14^ and almost absent in Japanese, Korean or Mongolian individuals.^15^, ^16^

Similarly, the frequency of mitochondrial mutations also differs among populations; the A1555G mutation is found mostly in patients with maternally inherited hearing loss, although it has been sporadically identified in patients with hereditary autosomal recessive conditions. There are major differences in ethnic origin in both transmission modes, as revealed by recent studies showing the mutation to be a frequent cause of non-syndromic hearing loss, associated or not with aminoglycoside use, in Asian,^11^, ^17^, ^18^, ^19^, ^20^, ^21^ Arab,^22^ and South African^23^ populations. It is, however, rare in most of the European and American populations.^24^, ^25^

A study in 32 Asians with hearing loss and in 100 American controls (63 deaf and 37 normal-hearing) found the A1555G mutation in 87.5% of Asians that were deaf, and in none of the subjects in the control group, concluding that the mutation is not prevalent in non-Asian populations.^25^ This results has been confirmed in a study done in Greece, in which the mutation was absent in 106 cases,^26^ and in another study involving 202 early onset non-syndromic hearing loss patients in the United Kingdom.5 Likewise in our study, the A1555G mitochondrial mutation was not found in the HLG groups (consisting of group I: 13 index cases that had taken aminoglycosides, and group II: 14 index cases that had no prior use of aminoglycosides) and in the control group (100 neonates with normal otoacoustic emissions tests). These results demonstrate that the mutation was not a common cause of hearing loss in our sample.

Two mutation screening program have been done in neonates in the USA. The first of these sampled umbilical cord blood of 1,773 neonates, of which only one carried the mutation.^27^ The second screened 25 neonates that had altered otoacoustic emissions tests, among which the mutation was not found.^28^ Screening of 300 neonates in Argentina and in 712 normal-hearing subjects did not detect the A1555G mutation.^29^ These studies have reported low rates in North-American populations, but have not discarded the need for molecular studies in neonatal screening tests, especially in countries where aminoglycosides are used routinely.

Our method has already been described in the literature;11 it includes amplifying a specific cytochrome b gene region - a highly conserved mitochondrial genome area - to serve as an amplification control for identifying mitochondrial DNA in all of the samples.^12^ Although the number of cases in both groups was different from the number in some published studies, and although the mutation was not found in both groups (HLG and control groups), we were able to confirm the results of studies that also did not find the mutation in non-Asian or Arab ethnic groups.^24^, ^25^, ^26^, ^27^, ^28^, ^29^

There is a single study in Brazil reporting the findings of five families with hearing loss cases in which the prevalence of the mutation was 2% (4 cases). Only one of these positive cases was associated with an assumed aminoglycoside exposure; no mutation was found in the control group composed by black, white or Asian (Japanese or Chinese) ethnic groups.^30^ These results - which are different from our study - may be explained by the highly heterogeneous ethnic composition of the Brazilian population that resulted from racial mixing; this may explain prevalence variations in different Brazilian regions.

Many mutations may result in hearing loss; the A1555G mitochondrial mutation, which is related to aminoglycoside use, may be only one of them.^17^, ^30^ Understanding the underlying causes of hearing loss caused by aminoglycoside exposure is essential for therapy - especially in neonatal ICUs - and for genetic counseling and early rehabilitation. This is necessary for including or returning these patients to their social and professional activities.

## CONCLUSION

The PCR/RFLP molecular techniques, such as the protocol we used in this study, are simple methods for tracking the A1555G mutation, supporting the molecular investigation of hearing loss.

There has been considerable interest in establishing the true prevalence of the A1555G mitochondrial mutation in the Brazilian population by means of molecular testing, as well as investigating other mutations that may result in hearing loss associated or not with aminoglycoside use. These tests may become a valuable adjunct for neonatal audiometric screening.
